# Prevalence of Primary HIV Drug Resistance in Thailand Detected by Short Reverse Transcriptase Genotypic Resistance Assay

**DOI:** 10.1371/journal.pone.0147945

**Published:** 2016-02-01

**Authors:** Sasisopin Kiertiburanakul, Subencha Pinsai, Wasun Chantratita, Ekawat Pasomsub, Manoon Leechawengwongs, Wilawan Thipmontree, Nirada Siriyakorn, Somnuek Sungkanuparph

**Affiliations:** 1 Department of Medicine, Faculty of Medicine Ramathibodi Hospital, Mahidol University, Bangkok, Thailand; 2 Department of Pathology, Faculty of Medicine Ramathibodi Hospital, Mahidol University, Bangkok, Thailand; 3 Vichaiyut Hospital, Bangkok, Thailand; 4 Department of Medicine, Maharat Nakhon Ratchasima Hospital, Nakhon Ratchasima, Thailand; University of Athens, Medical School, GREECE

## Abstract

**Background:**

HIV drug resistance (HIVDR) is the major cause of treatment failure after scaling up of antiretroviral therapy (ART). HIVDR testing prior to ART initiation is not routinely performed in resource-limited settings. We aimed to assess the prevalence of primary HIVDR by short reverse transcriptase (RT) genotypic resistance assay and evaluate of the impact of the mutations on the treatment outcomes.

**Methods:**

A prospective cohort study was conducted in treatment-naïve HIV-infected patients. Fourteen major mutations of codon 99–191 on the RT gene were selected (K103N, V106A/M, V108I, Q151M, Y181C/I, M184V/I, Y188C/L/H, and G190S/A) at a cost of testing of 35 USD. The association between the presence of primary HIVDR and undetectable HIV RNA (<50 copies/mL) after 6 months of ART was determined.

**Results:**

A total of 265 HIV-infected patients were included, with a median age of 35.2 (range, 16.8–75.2) years; 62.6% were males. The median (interquartile range) CD4 cell count at ART initiation was 216 (77–381) cells/mm^3^. The overall prevalence of primary HIVDR was 7.9%. The prevalence of each HIVDR mutation were K103N 6.0%, V106I 1.1%, V108I 0.4%, Y181C 2.3%, Y181I 0.7%, Y181V 0.4%, M184V 3.0%, M184I 1.5%, and G190A 2.3%. No associated factor of having primary HIVDR was determined. By multiple stepwise logistic regression, factors associated with undetectable HIV RNA after 6 months of ART were: having M184V/I (odds ratio [OR] 0.11; 95% confidence interval [CI] 0.02–0.62, *p* = 0.013), condom use (OR 2.38; 95% CI 1.12–5.06, *p* = 0.024), and adherence per 5% increase (OR 1.16; 95% CI 1.00–1.35, *p* = 0.044).

**Conclusions:**

The prevalence of primary HIVDR is approximately 8%; it is associated with detectable HIV RNA at 6 months after ART initiation. Routine “short RT” genotypic resistance assay should be considered in resource-limited settings to maximize treatment outcome.

## Introduction

In 2001, the Thai government started the National Access to Antiretroviral Program for People Living with HIV/AIDS, which provides antiretroviral therapy (ART) free of charge. Access to ART has dramatically expanded; by the end of 2011, 225,272 people in Thailand had received ART [1]. The results of some studies among HIV-infected Thai patients have demonstrated a reduction in AIDS-related mortality and morbidity by ART [2–4]. In spite of the success of ART, HIV drug resistance (HIVDR) is the major cause of treatment failure after scaling up of ART [5–6].

Primary HIVDR means that there is resistance of HIV to antiretroviral drugs seen in individuals who have never received ART and who presumably have been infected with a drug-resistant virus [7–9]. Primary HIVDR is increasing; the reported prevalence varies from approximately 1.1% to 21% in the United States, Europe, and Africa [10–16]. Resistance mutations to non-nucleoside reverse transcriptase inhibitors (NNRTIs) are also highly prevalent and increasing in some areas, reflecting local ART prescription patterns [17,18]. Recently, two studies among treatment-naive HIV-infected Thai patients demonstrated that the prevalence of NRTI, NNRTI, and protease inhibitor (PI) drug resistance mutations were 0.6% and 1.9%, 17% and 2.8%, and 0.6% and 1.7%, respectively [10,17,19].

The potency of ART may be reduced in patients who have acquired a resistant virus. Accordingly, several studies in developed countries have shown a reduction in the efficacy of ART in patients with primary HIVDR compared with patients without primary HIVDR [13,15,18,20,21]. As a result, HIV RNA decline, the time required to reach suppression of viral replication, and the percentage of patients with undetectable HIV RNA were inferior in patients with primary HIVDR [15,18,21,22–24].

Currently, HIVDR testing prior to ART initiation is not routinely recommended in resourced-limited countries, including Thailand [25,26], due to lack of infrastructure, lack of cost-effectiveness studies, previously low reported prevalence of HIVDR, and limited number of studies regarding treatment outcome among patients with primary HIVDR. We hypothesized that genotypic sequencing of HIV reverse transcriptase (RT) as a short sequence (codon 99–191), namely “short RT” method, could detect the majority of HIVDR mutations. The cost of this “short RT” method is lower than the standard genotypic resistance testing, approximately 35 USD, and hence might be more cost-effective than full-length genotype sequencing. Thus, the objectives of this study were: to evaluate the prevalence of emerging HIVDR by “short RT” genotypic resistance assay in ART-naive HIV-infected Thai patients who were about to initiate first-line ART; to determine the factors associated with having primary HIVDR; and to determine the effect of primary HIVDR on treatment outcome after 6 months of ART.

## Methods

A prospective cohort study was conducted in patients who were diagnosed with HIV infection and who were about to initiate ART from August 2011 to June 2014. Patients from hospitals throughout Thailand were enrolled in the study. Inclusion criteria were patients with a confirmed diagnosis of HIV infection by positive HIV testing according to the Thailand National Guidelines on HIV/AIDS Treatment and Prevention [25], and who were naïve to ART. Patients with a history of exposure to antiretroviral drugs, including mono or dual therapy, or prevention of mother-to-child transmission were excluded. Blood samples drawn from patients before ART initiation were sent to the virology and molecular biology unit, Department of Pathology, Faculty of Medicine Ramathibodi Hospital, for detection of primary HIVDR. Ethical approvals of this study were obtained from committee on human rights related to research involving haman subjects, Faculty of Medicine Ramathibodi Hospital, Mahidol University (approve number: ID 08-56-20). All patients provided written informed consent prior to to participate in this study.

A short version of the genotypic resistance assay was designed for detection of codon 99–191 on the RT gene. This region was selected to cover 14 mutations (K103N, V106A/M, V108I, Q151M, Y181C/I, M184V/I, Y188C/L/H, and G190S/A) contributing to NRTI and/or NNRTI resistance. The “short RT” genotypic resistance assay is briefly described as follows. Plasma HIV RNA was purified and extracted using a NucliSENS^®^ EasyMAG automatic system (bioMérieux, Marcy l'Etoile, France). The total RNA was eluted into 55 μL. The reverse transcription–polymerase chain reaction (PCR) of the specific mutation of RT was performed using an Invitrogen SuperScript^®^ RT-PCR System with Platinum *Taq* DNA Polymerase, with primer pairs HIV-RTF1 (AgACCAgAgCCAACAgCC) and HIV-RTR1 (CTTgCCCAATTTAgTTTTCC). The master mix for RT-PCR contained 25 μL reaction mix (2x), 2.5 μL HIV-RTF1 primer (10 μM), 2.5 μL HIV-RTR1 primer (10 μM), 1 μL RT *Taq* enzyme, 1.5 μL MgSO_4_ (50 mM), and 12.5 μL distilled water. A total volume of 45 μL of master mix was mixed with 5 μL of RNA template. Amplification was performed in a thermal cycler. Purification was performed with a MinElute PCR Purification Kit (Qiagen, Hilden, Germany), and then was run on gel (1 kb) for about 1 h. The PCR cleanup process contained PCR product of 5 μL and USB ExoSAP-IT reagent (Affymetrix, USA) of 2 μL. The product was then mixed, incubated at 37°C for 15 min, and inactivated at 80°C for 15 min. PCR products corresponding to the RT coding regions were sequenced using an Applied Biosystems 3500 series genetic analyzer (8-capillary array). The master mix for sequencing contained 4.0 μL BigDye^®^ Terminator (v.3.1), 2.0 μL sequencing buffer (5x), 0.5 μL primer (10 mM), 6.5 μL distilled water, and 7.0 μL template. In total, the volume of master mix was 20 μL. The sequencing results were edited using the BioEdit sequence alignment editor and analysis program (Tom Hall, Ibis Biosciences, Carlsbad CA, USA).

HIVDR-associated mutations were interpreted to the levels of susceptibility to the RT gene by the Stanford Genotypic Resistance Interpretation Algorithm (HIValg) from the Stanford University HIV Drug Resistance Database [27]. Medical records were retrieved and reviewed. The following variables were collected: (1) clinical characteristics, including gender, age, routes of HIV transmission, prior AIDS-defining illness, underlying associated condition, ART regimen, HIV prevention method, and contraception; (2) laboratory-related data, including CD4 cell count and HIV RNA; (3) Adherence and adherence percentage (determined by pill count); and (4) partner’s information, such as HIV treatment, ART regimen, current CD4 cell count, and HIV RNA.

Mean with standard deviation (SD), median with interquartile range (IQR), and frequency (%) were used to describe patients’ characteristics. Categorical variables between the two groups were compared using chi-square or Fisher’s exact test, as appropriate. Continuous variables between the two groups were compared using Student’s *t*-test and Mann–Whitney *U* test, as appropriate. The association between the presence of primary HIVDR and undetectable HIV RNA (<50 copies/mL) after 6 months of ART was determined by logistic regression. Variables that presented *p* <0.10 were considered in a multivariate logistic regression model after assessment of multicollinearity using variance inflation factors. Variables were selected out from a multiple logistic regression model with backward stepwise selection; ones that attained a level of significance were retained in the model. A *p* <0.05 was considered statistically significant. All statistical analyses were performed using Stata statistical software version 12.0 (StataCorp, College Station TX, USA).

## Results

**Fig 1:**
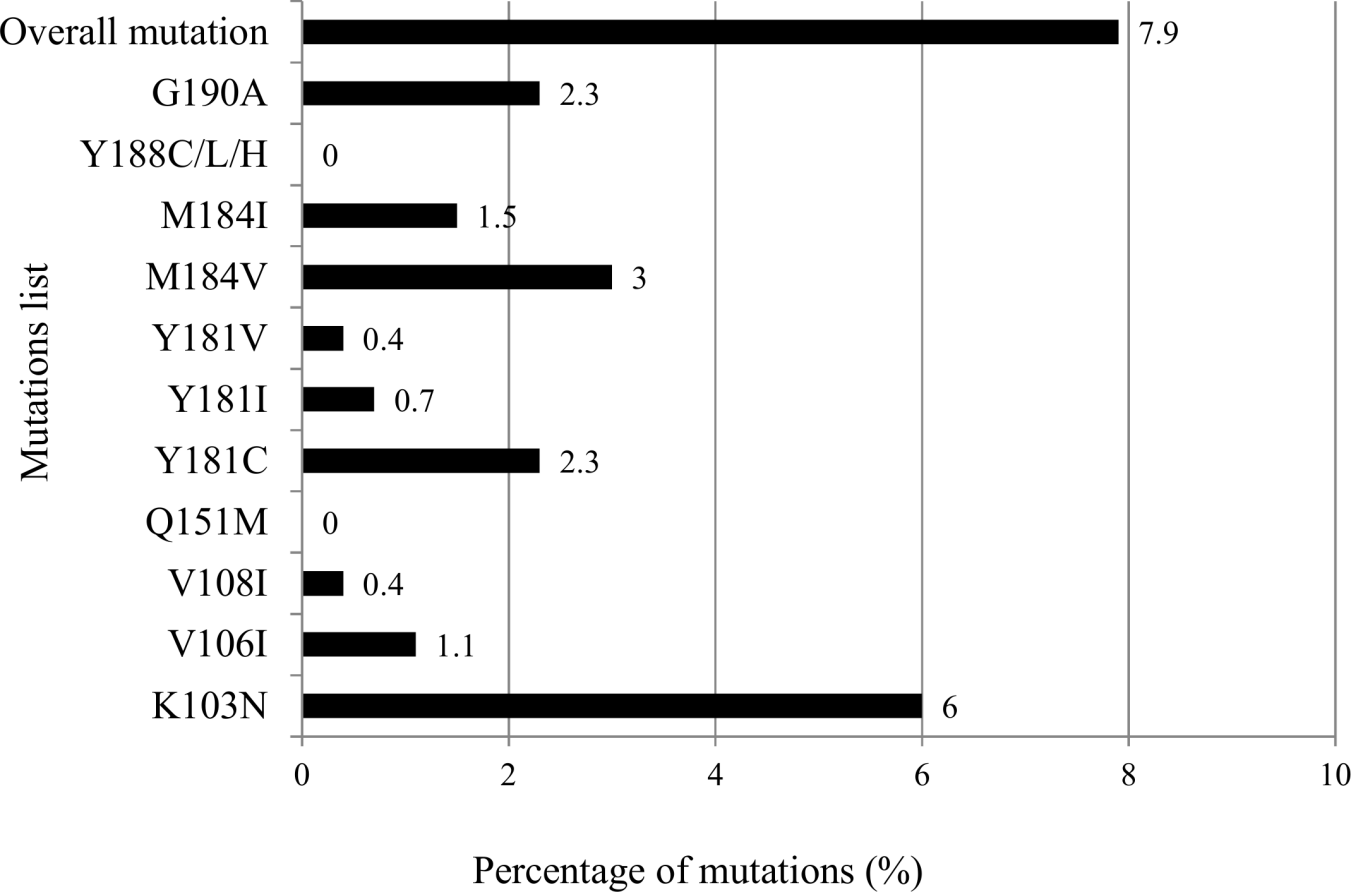
Prevalence of primary HIV drug resistance mutations, from short reverse transcriptase genotypic resistance assay.

A total of 265 naïve HIV-infected patients were included; their baseline characteristics are shown in Table 1. The overall prevalence of primary HIVDR was 7.9%. The prevalence of each mutation was: K103N (6.0%), M184V (3.0%), G190A (2.3%), Y181C (2.3%), M184I (1.5%), V106I (1.1%), Y181I (0.7%), V108I (0.4%), and Y181V (0.4%). No patients had the mutations Y188C/L/H and Q151M. The prevalence of HIVDR mutations is shown in Fig 1.

**Table 1:** Demographic and Baseline Characteristics of 265 Treatment-Naïve HIV-Infected Patients.

Variables	Values
Median (range) age at HIV diagnosis, years	35.2 (16.8–75.2)
Gender, n (%)	
Male	166 (62.6)
Female	99 (37.4)
Route of HIV transmission, n (%)	
Heterosexual	168 (63.4)
Homosexual	80 (30.2)
Intravenous drug use	4 (1.5)
Blood transfusion	1 (0.4)
Other	8 (3.0)
Unknown	4 (1.5)
Opportunistic infection or AIDS-defining illness, n (%)	
No	126 (47.6)
Yes	139 (52.4)
Tuberculosis	67 (25.3)
*Pneumocystis jirovecii* pneumonia	27 (10.2)
Cryptococcosis	6 (2.3)
Cytomegalovirus disease	4 (1.5)
Malignancy	2 (0.7)
Others	33 (12.4)
HIV prevention and/or contraception, n (%)*	
No prevention	80 (30.2)
Condom use	175 (66.0)
Oral contraceptive drugs	21 (7.9)
Not available	10 (3.8)
At HIV diagnosis	
Median (IQR) CD4 cell count, cells/mm^3^^†^	292 (87–466)
Median (IQR) CD4 cell percentage, %	14.5 (6–20)
At ART initiation	
Median (IQR) CD4 cell count, cells/mm^3^^‡^	216 (77–381)
Median (IQR) CD4 cell percentage, %	13 (6–18)
Median (IQR) HIV RNA, copies/mL^§^	65,700 (17,306–211,256)

IQR, interquartile range.

*Some patients might use more than one method.

^†^CD4 cell count at HIV diagnosis was available in 260 patients.

^‡^CD4 cell count at ART initiation was available in 220 patients.

^§^HIV RNA at ART initiation was available in 117 patients.

The comparison of characteristics between patients with and without primary HIVDR is summarized in Table 2. Patients with primary HIVDR had a lower median CD4 cell count at ART initiation (76 vs. 232 cells/mm^3^, *p* = 0.010). By univariate logistic regression analysis, only CD4 cell count <200 cells/mm^3^ at ART initiation was a factor significantly associated with having primary HIVDR (odds ratio [OR] 0.28; 95% confidence interval [CI] 0.87–0.90, *p* = 0.032). However, no factor was associated with having primary HIVDR, as determined by multivariate logistic regression analysis.

**Table 2:** Comparison of Clinical Characteristics, Laboratory Investigations, and Clinical Outcomes between Patients with and without Primary HIV Drug Resistance.

Variables	Patients with Primary HIVDR (n = 21)	Patients without Primary HIVDR (n = 244)	*P*-value
**Clinical Characteristics**			
Median (range) age at time of HIV diagnosis, years	38 (30–47)	36 (30–43)	0.491
Gender			0.816
Male	14 (66.7)	152 (62.3)	
Female	7 (33.3)	92 (37.7)	
Route of HIV transmission, n (%)			0.681
Heterosexual	16 (76.2)	152 (62.3)	
Homosexual	4 (19.0)	76 (31.2)	
Intravenous drug use	0 (0)	4 (1.6)	
Other	1 (4.8)	7 (2.9)	
Condom use, n (%)	15 (71.4)	160 (65.6)	0.641
Partner on ART, n (%)	6 (28.6)	47 (19.9)	0.397
**Laboratory investigations**			
Median (IQR) CD4 cell count at HIV diagnosis, cells/mm^3^*	193 (31–515)	295 (93–466)	0.355
Median (IQR) CD4 cell count at ART initiation, cells/mm^3^^‡^	76 (22–228)	232 (86–391)	0.010
Median (IQR) HIV RNA at ART initiation, copies/mL^†^	88,403 (2,888–21,1256)	64,450 (21,401–216,003)	0.588
Median (IQR) CD4 cell count of partner, cells/mm^3^^§^	304 (135–410)	401 (206–468)	0.356
Partner with undetectable HIV RNA, n (%)^||^	4/4 (100)	16/22 (72.7)	0.234
**Clinical outcomes**			
Median (IQR) CD4 cell count at 6 months after ART initiation, cells/mm^3^^¶^	232 (48–337)	281 (225–533)	0.016
HIV RNA <50 copies/mL at 6 months after ART initiation, n (%)^#^	7/13 (53.9)	141/178 (79.2)	0.077

ART, antiretroviral therapy; HIVDR, HIV drug resistance; IQR, interquartile range.

*CD4 cell count at HIV diagnosis was available in 260 patients.

^‡^CD4 cell count at ART initiation was available in 220 patients.

^†^HIV RNA at ART initiation was available in 117 patients.

^§^CD4 cell count of partner was available in 38 partners.

^||^HIV RNA of partner was available in 26 partners.

^¶^CD4 cell count at 6 months after ART initiation was available in 193 patients.

^#^HIV RNA at 6 months after ART initiation was available in 191 patients.

Fifty-six patients were lost to follow-up after HIV diagnosis and the information regarding ART initiation was missing. A total of 209 patients (78.9%) received ART and were included in the following part of the analysis. The five most common antiretroviral regimens were: tenofovir disoproxil fumarate (TDF)/emtricitabine (FTC)/efavirenz (EFV), 24.1%; TDF/lamivudine (3TC)/EFV, 20.8%; zidovudine (AZT)/3TC/nevirapine (NVP), 9.4%; AZT/3TC/EFV, 7.6%, and stavudine/3TC/NVP, 6.4%. After 6 months of ART initiation, patients with primary HIVDR had a lower median CD4 cell count (232 vs. 281 cells/mm^3^, *p* = 0.016) and a lower proportion of HIV RNA <50 copies/mL (53.9% vs. 79.2%, *p* = 0.077). By univariate logistic regression, CD4 cell count <200 cells/mm^3^ at ART initiation (OR 2.03; 95% CI 1.01–4.09, *p* = 0.047), adherence per 5% increase (OR 1.22; 95% CI 1.07–1.39, *p* = 0.004), having primary HIVDR (OR 0.31; 95% CI 0.10–0.97, *p* = 0.043), having M184V/I (OR 0.08; 95% CI 0.02–0.44, *p* = 0.003), having G190S/A (OR 0.09; 95% CI 0.01–0.90, *p* = 0.040), and having Y181C/I (OR 0.07; 95% CI 0.01–0.61, *p* = 0.017) were significantly associated with HIV RNA <50 copies/mL after 6 months of ART (Table 3). By multiple stepwise logistic regression, having M184V/I (OR 0.11; 95% CI 0.02–0.62, *p* = 0.013), condom use (OR 2.38; 95% CI 1.12–5.06, *p* = 0.024), and adherence per 5% increase (OR 1.16; 95% CI 1.00–1.35, *p* = 0.044) were associated with HIV RNA <50 copies/mL after 6 months of ART.

**Table 3:** Factors Associated with Undetectable HIV RNA at 6 Months after ART Initiation, by Univariate Logistic Regression.

Factors	Odds Ratio	95% Confidence Interval	*P*-value
Age	0.98	0.95–1.01	0.267
Female	0.87	0.44–1.73	0.693
Heterosexual	0.53	0.25–1.14	0.105
Had AIDS-defining illness	0.73	0.36–1.45	0.369
Condom use	1.83	0.90–3.73	0.097
CD4 cell count <200 cells/mm^3^ at HIV diagnosis	1.64	0.83–3.24	0.159
CD4 cell count <200 cells/mm^3^ at ART initiation	2.03	1.01–4.09	0.047
HIV RNA at ART initiation, per log copies/mL	0.97	0.57–1.67	0.918
Having primary HIVDR	0.31	0.10–0.97	0.043
Having K103N	1.17	0.13–10.72	0.892
Having M184V/I	0.08	0.02–0.44	0.003
Having G190S/A	0.09	0.01–0.90	0.040
Having Y181C/I	0.07	0.01–0.61	0.017
Adherence, per 5% increase	1.22	1.07–1.39	0.004
Partner on ART	1.18	0.49–2.80	0.713
Partner had undetectable HIV RNA	1.01	0.98–1.03	0.710

ART, antiretroviral therapy; HIVDR, HIV drug resistance.

## Discussion

The present study showed an overall prevalence of primary HIVDR of approximately 8% by “short RT” genotypic resistance assay among naïve HIV-infected Thai patients. This number is categorized as moderate prevalence by the World Health Organization (WHO) criteria [8]. In recent studies, the prevalence of primary HIVDR has ranged from 0% to 17.6% in Thailand and from 0% to 13.8% in Asian countries [11,18,19, 28–33]. A recent study reported that the prevalence of transmitted HIVDR in Asia was 4.2% and the prevalence of NRTI and NNRTI resistance had risen significantly over time [34]. The results of these studies have varied depending on study methodology, mode of HIV transmission of the enrolled patients, duration of HIV infection, HIV subtype, pattern of local ART regimen use, and the reference list of HIVDR used to assess the presence of HIVDR mutations [33].

In Thailand, the prevalence of primary HIVDR for NRTI was 0.6–1.9% and for NNRTI 2.8–17% [10,17,19,28]. M184V/I and Y181C are the common mutations of primary HIVDR reported in Thailand [10,17,19,28]. K103N was the most common mutation detected in the present study, with a prevalence of 6%. This mutation is usually selected by NVP and/or EFV, the most widely used NNRTI in resource-limited countries, including Thailand [25,26,35]. M184I/V was the second most common mutation (4.5%) detected in the present study. This mutation is selected by 3TC or FTC, which is one of the important backbones in the first-line ART regimens recommended by all guidelines [9,25,26,35]. These mutations could be detected by the “short RT” genotypic resistance assay that we developed to cover the most common and significant mutations in Thailand. Similar to previous findings in Thai patients, no factor significantly associated with primary HIVDR was identified in the present study [17]. This might be due to the relatively low number of patients with primary HIVDR in our cohort.

Only a limited amount of data has been available regarding primary HIVDR and response to ART in resource-limited settings. Multicenter cohort studies have demonstrated that primary HIVDR is associated with poor treatment outcomes and/or clinical complications in developed countries [36–39]. We found that having primary HIVDR is a crucial factor in determining the therapy response, as in the recent study conducted in the Southeast Asia region [40]. In addition, lower treatment adherence and not using a condom were significantly associated with undetectable HIV RNA after 6 months of ART in our patients. We hypothesized that not using a condom might accelerate the risk of new HIV strain including HIV resistance strain transmission.

The strength of this study is that it was a multi-center study; patients were enrolled from hospitals countrywide. The prevalence of approximately 8% should be a representative value of primary HIVDR in Thailand. Furthermore, we developed a new method and/or concept of primary HIVDR detection, the “short RT” genotypic resistance assay, to retain the efficiency of major resistance mutation detection while lowering the cost, thus making the test more accessible. The short RT is a simple in-house sequencing. To minimize the cost, we designed PCR and sequencing premiers to sequence only part of the pol gene. For the sequencing part, we employed a standard protocol for the conventional sequencing. This in-house short RT sequencing method is an alternative method for core facilities either resource-rich and resource-limited countries that currently use NNRTI-based regimens. Therefore, any resource-limited countries that have used either US FDA-approved HIV-1 DR genotyping system, in-house, or “home-brew” genotyping systems could easily utilize the short RT method. Lastly, this study is one of the largest to demonstrate the effect of primary HIVDR on treatment outcome.

There are some limitations of our study. First, the patients were tested for HIV genotypes at pretreatment rather than at the time of diagnosis. Thus, some mutations may have reversed to the wild type, and the prevalence of primary HIVDR detected by “short RT” genotypic resistance assay could be underestimated. The prevalence of primary HIVDR among patients with recent HIV infection and those with chronic HIV infection might be different [30]. Second, approximately 9% of the patients (19 of 209 whom ART were initiated) were lost to follow-up after ART initiation, and we were unable to evaluate their treatment outcome. Third, the number of patients with primary HIVDR was relatively too small to determine the effect of the relevant mutations other than M184V/I on the treatment outcomes. Lastly, the “short RT” assay that we used in this study might underestimate the magnitude of primary HIVDR if some mutations were out of the codons of “short RT” that we designed. Nevertheless, using this assay is far better than not testing for primary HIVDR in resource-limited settings.

## Conclusions

A moderate level of primary HIVDR was determined after a decade of rapid scaling up of ART in Thailand. Primary HIVDR was detected in approximately 8% of naïve HIV-infected Thai patients by a low-cost, “short RT” genotypic resistance assay. The presence of primary HIVDR, especially having M184I/V, is associated with detectable HIV RNA at 6 months after ART initiation. Routine “short RT” genotypic resistance assay for detection of primary HIVDR should be considered in Thailand to maximize treatment outcome.
